# Prevalence of Binge Drinking by Caregivers of Persons with Alzheimer’s Disease or Related Dementia

**DOI:** 10.1093/geroni/igab046.2985

**Published:** 2021-12-17

**Authors:** Zachary Kunicki, Richard Jones

**Affiliations:** Brown University, Providence, Rhode Island, United States

## Abstract

Some caregivers of persons with Alzheimer’s Disease and related dementias (ADRD) are known to be under high levels of burden, which is associated with higher levels of anxiety, depression, and stress. Previous research has established anxiety, depression, and stress are associated with binge drinking, but little research has examined binge drinking rates among ADRD caregivers. Binge drinking could influence the ability of ADRD caregivers to provide care. The purpose of this study was to explore the prevalence and prevalence correlates of binge drinking among ADRD caregivers using the 2019 Behavior Risk Factor Surveillance Survey (BRFSS). We identified N = 1,642 persons who were the primary informal caregivers of a person with ADRD. Among them, the prevalence of binge drinking was 14 per 100 persons. Bivariable analyses suggested male caregivers and caregivers with 14 or more days of poor mental health in the past 30 days had the highest prevalence of binge drinking at 18 per 100 persons. Caregivers who were 65 or older or had the lowest prevalence at 3 per 100 persons. Caregiving characteristics revealed providing 20 to 39 hours of care per week had the highest prevalence of binge drinking (17 per 100) whereas spousal caregivers (9 per 100) had the lowest prevalence. Smoking status and hours per week providing care were associated with higher odds of binge drinking in multivariable analyses. Future research should examine if binge drinking by ADRD caregivers is related caregiver burden and the quality of care provided to the persons with ADRD.

